# Supplementary material for: Intravital imaging by simultaneous label-free autofluorescence-multiharmonic microscopy
Source: Nat Commun. 2018 May 29;9:2125. doi: 10.1038/s41467-018-04470-8 (PMC5974075; doi:10.1038/s41467-018-04470-8)
Supplement: Supplementary file 1 — Supplementary Information [file 41467_2018_4470_MOESM1_ESM.pdf]

Intravital imaging by simultaneous label-free autofluorescence-multiharmonic (SLAM) microscopy

You et al.

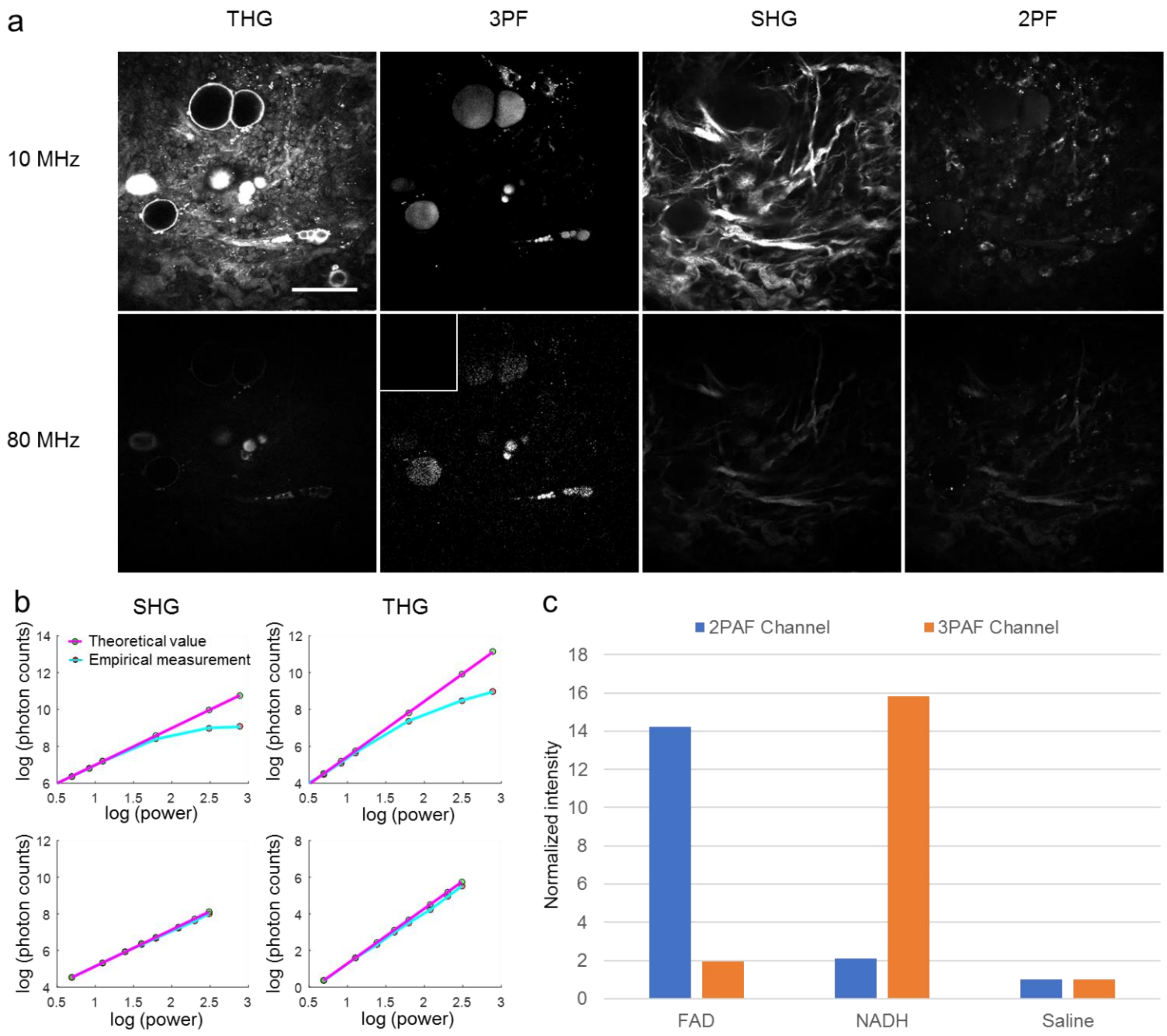

Supplementary Figure 1. Comparison of the signal generation efficiency between 10 MHz and 80 MHz repetition rate lasers. (a) Images acquired from the same site by excitation of the different repetition rates using the same average power of 8 mW after the objective. The contrast adjustment remained the same for each modality between the two laser sources except for the image of 3PAF acquired from 80 MHz. The raw image is displayed by the inset image in the left corner of the adjusted image. (b) Qualitative comparison of the signal generation efficiency between the 10 MHz (top row) and 80 MHz (bottom row) lasers. The photon counts were the average value of the 100 strongest data points measured from each image. The theoretical values were obtained from a second/third-order model for SHG/THG processes. (c) Signal analysis from an FAD solution, an NADH solution, and saline (control). The intensity was computed by averaging the pixel values of the image obtained from different solutions and then normalized against the signals from saline in corresponding channels. Scale bar: 50 microns.

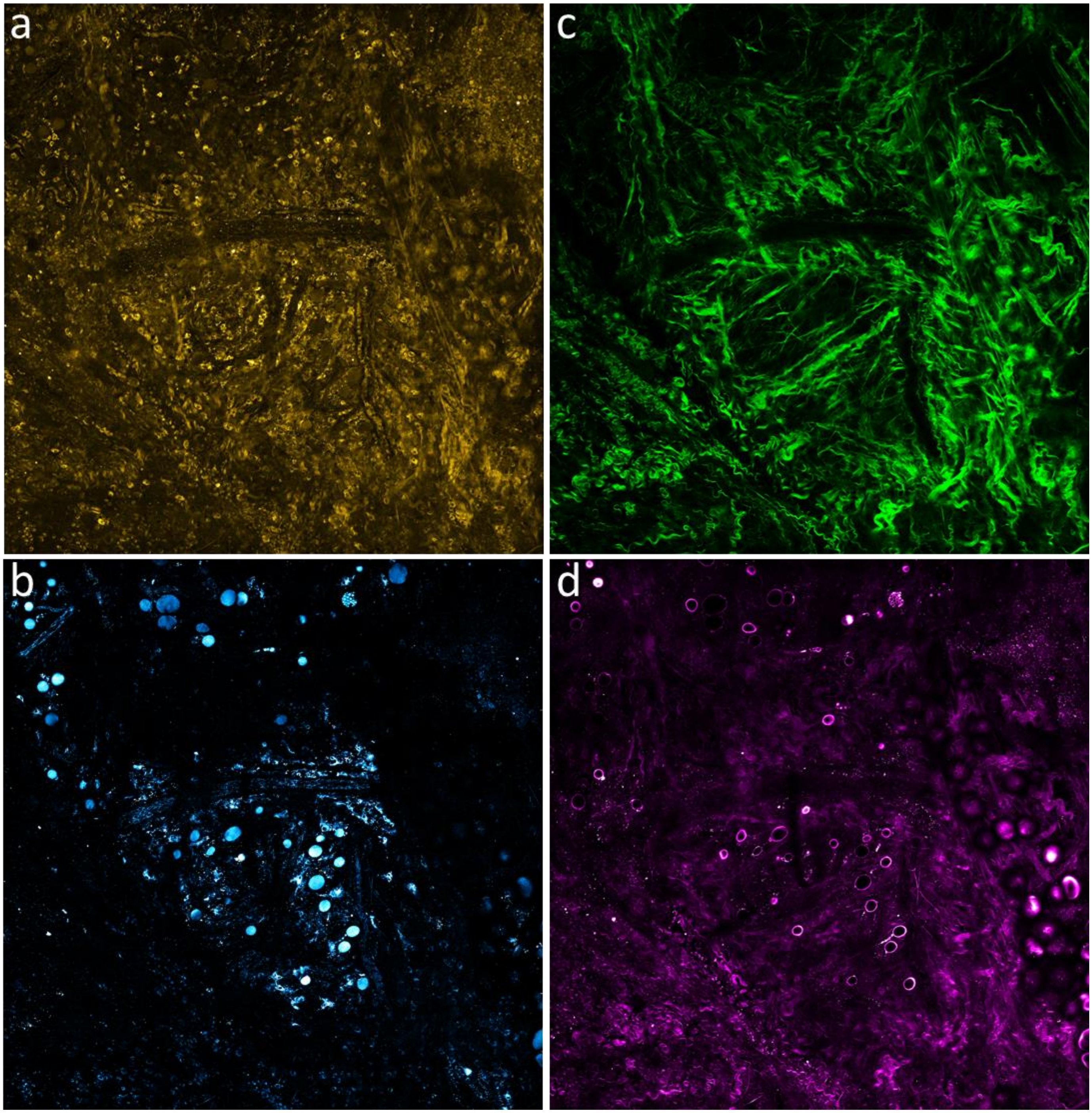

Supplementary Figure 2. Individual channels of Fig. 2a. (a) 2PAF channel that shows perfusing vasculature, stromal cells, and elastin fibers. (b) 3PAF channel that shows lipids, vesicles, endothelial cells, and macrophages. (c) SHG channel that shows collagen fibers. (d) THG channel that shows the lipid-water and protein-water interfaces, such as the boundaries of lipid droplets and vesicles near tumor cells.

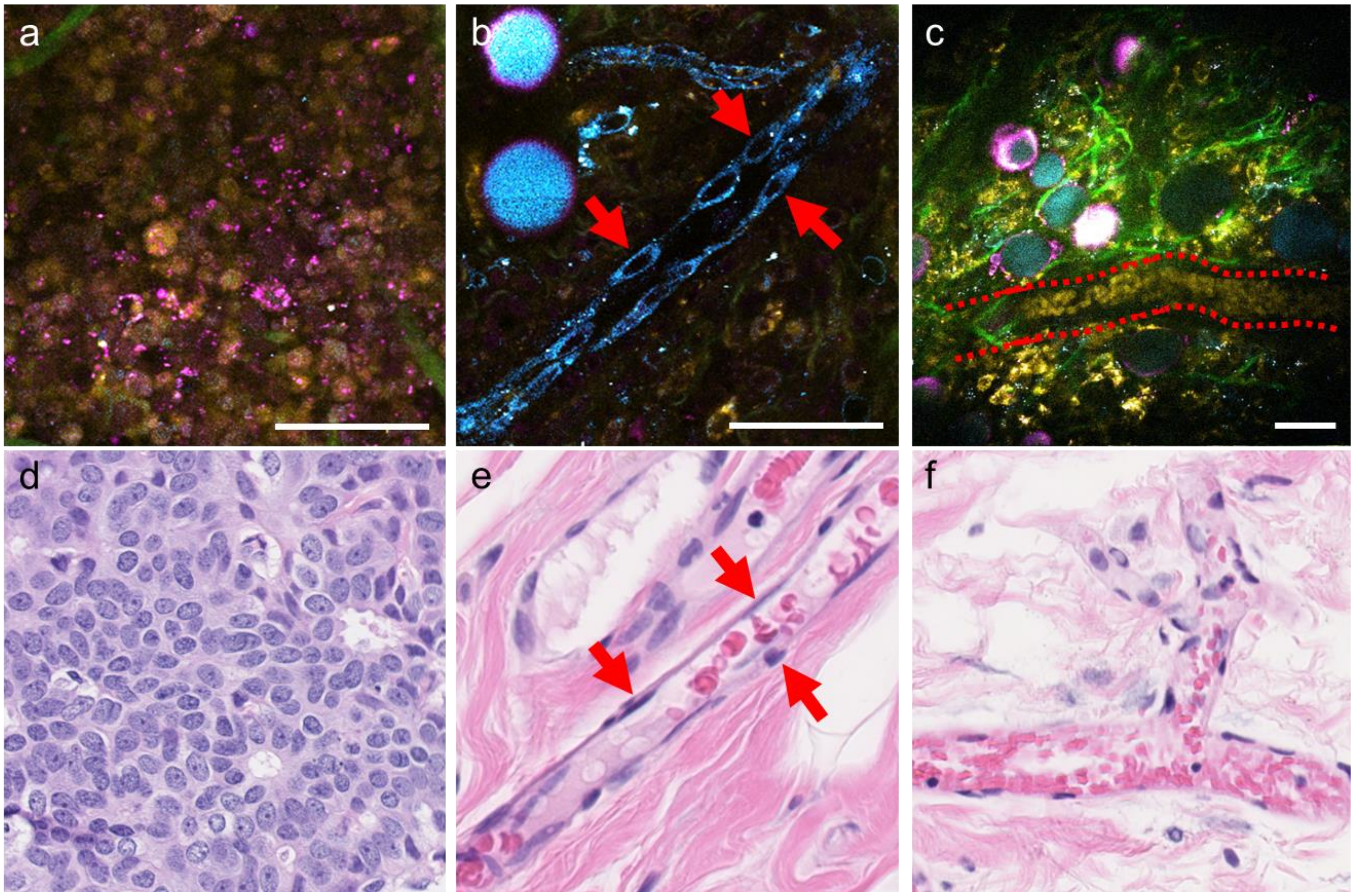

Supplementary Figure 3. Images of tumor cells, endothelial cells, and red blood cells by SLAM microscopy (a-c) and corresponding H&E-stained histology (d-f). Red arrows in (b,e) point to corresponding cellular features, and dotted red lines in (c) outline the walls of a blood vessel. Scale bar: 50 µm.

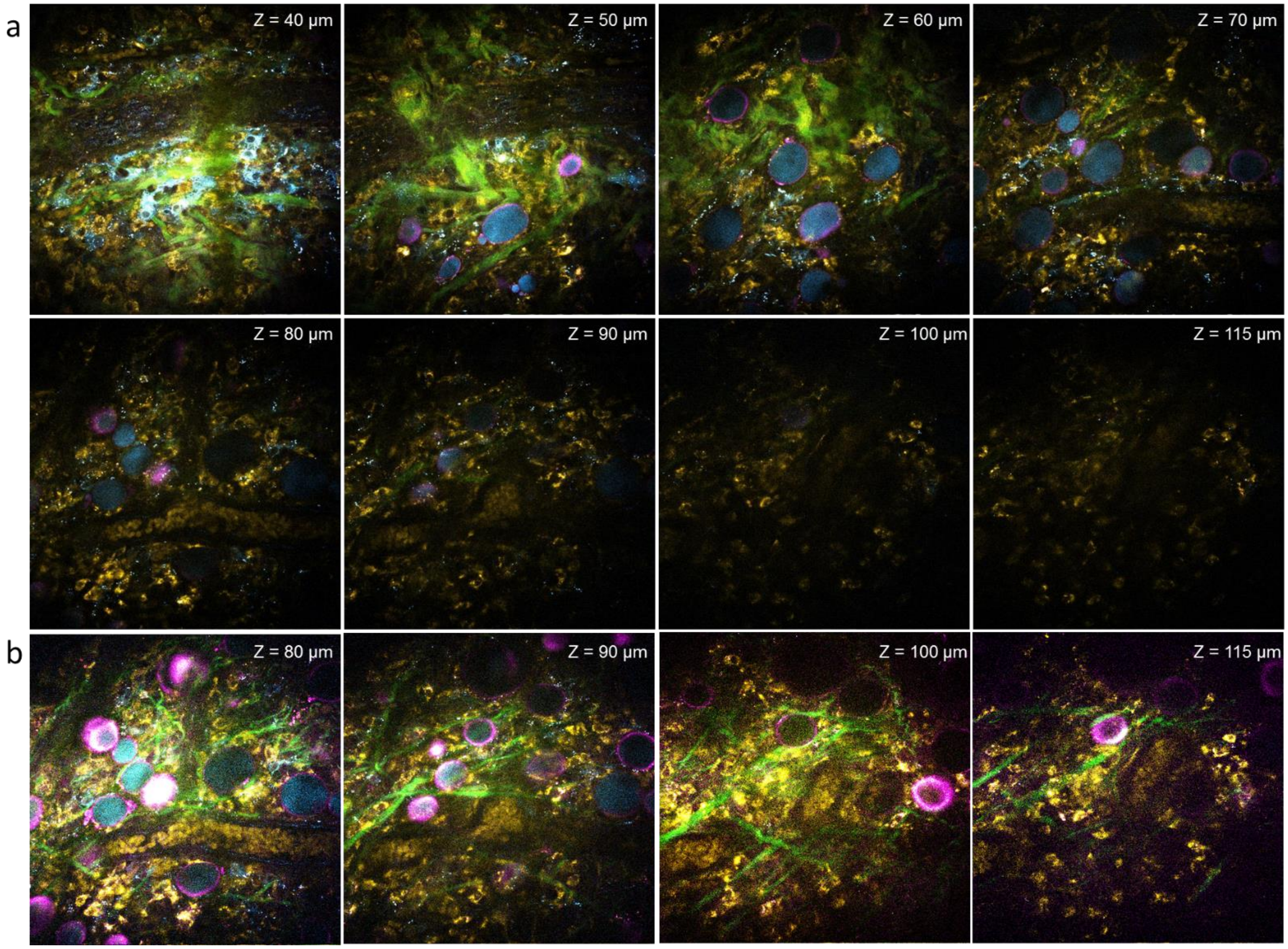

Supplementary Figure 4. Representative images at different depths within the tumor microenvironment (Fig2. f-h and Supplementary Movie 1). (a) Images with consistent contrast throughout the depth. (b) In order to better visualize structures, contrast and brightness were increased (FIJI, National Institutes of Health) for images acquired at deeper locations.

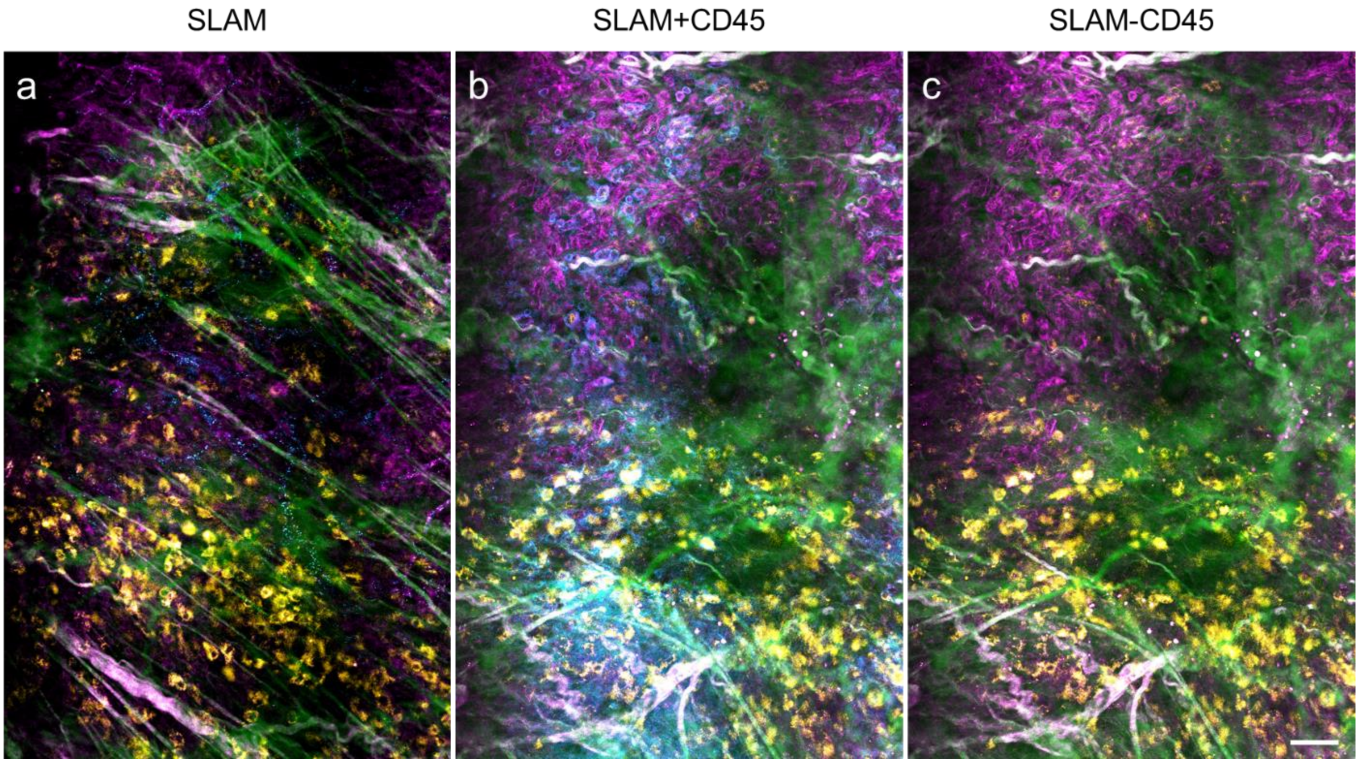

Supplementary Fig. 5. SLAM microscopy of the tumor microenvironment of a living rat before (a) and after (b,c) CD45 staining. The blue channel was assigned to NADH before staining and assigned to CD45 after staining. The images after staining (with or without CD45 channel) show cells with overlapping THG (magenta) and CD45 (blue) signals, demonstrating the specificity of THG signals. Scale bar: 50 µm. More detailed description can be found in Supplementary Note 3.

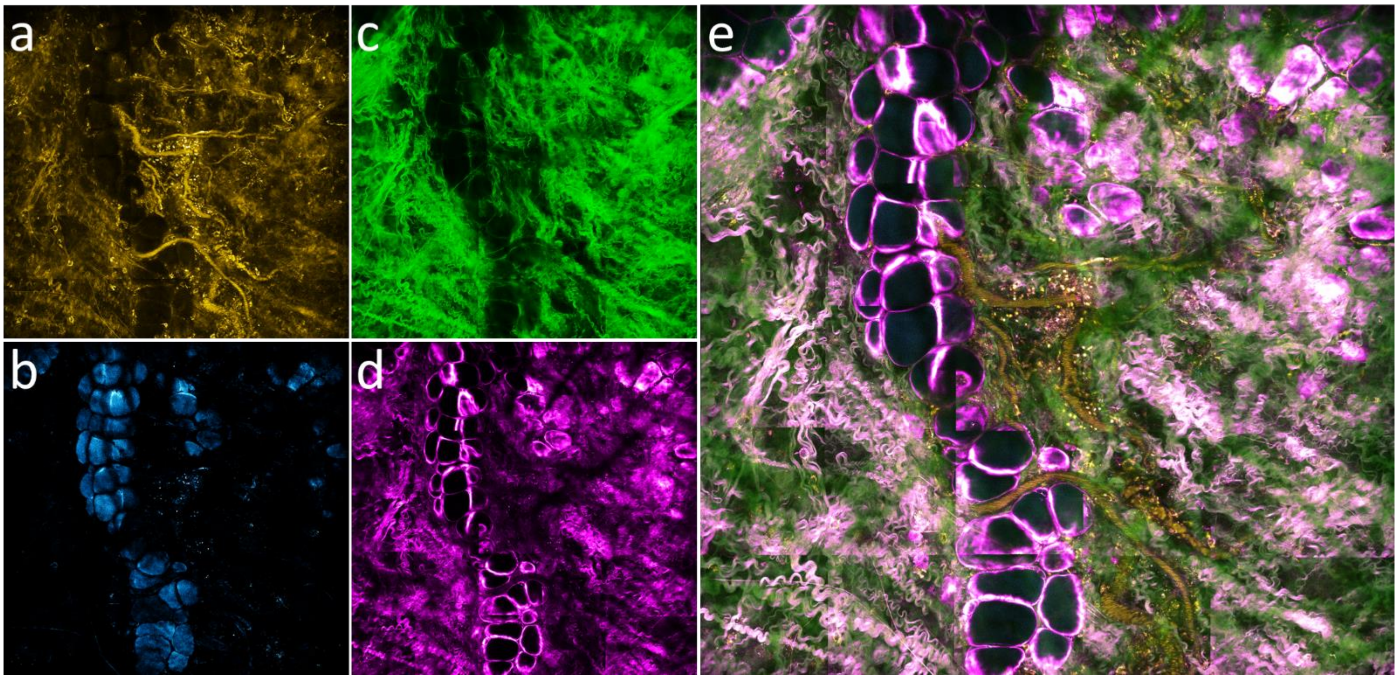

Supplementary Figure 6. Mosaic image collected surrounding the site in Fig. 4. (a) 2PAF channel that shows the perfusing vasculature, stromal cells, and elastin fibers. (b) 3PAF channel that shows lipids and vesicles. (c) SHG channel that shows collagen fibers. (d) THG channel that shows the lipid-water and protein-water interfaces, such as the boundaries of lipid droplets and the granules inside the leukocytes. (e) Composite of the previous four channels. Field of view: 1.1 mm x 1.1 mm.

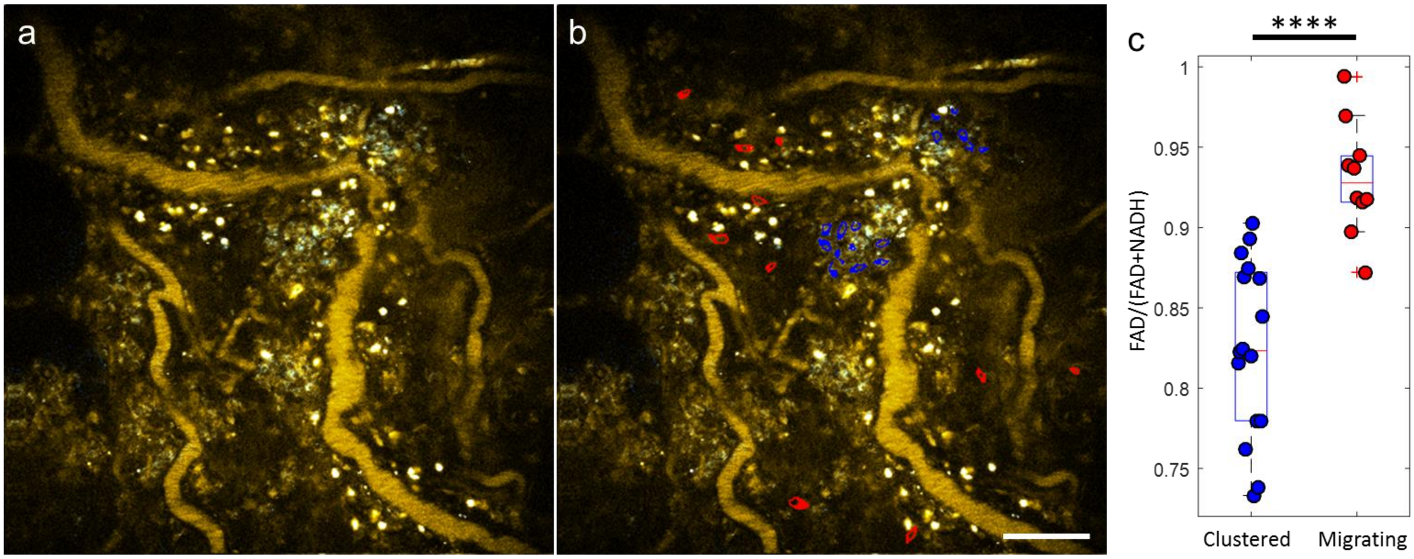

Supplementary Figure 7. Metabolic profiling of cells with different motion dynamics. (a) Image (2PAF (yellow) and 3PAF (blue)) was extracted from the end of Supplementary Movie 5 and shows clustered leukocytes and migrating leukocytes at the same site. (b) A mask marks two sets of representative cells – clustered leukocytes (blue) and migrating leukocytes (red). (c) Scatterplot of the redox ratio for each cell in the clustered group and the migrating group. The redox ratio significantly ($p<0.0001$ by Student's t-test) decreased in the clustered cells compared to the migrating cells. Scale bar: 50 µm.

Supplementary Note 1: Optimization of the SLAM condition.

To serve as a practical microscope in modern biology laboratories, SLAM microscopy should be optimized and potentially standardized so that similar microscopes across different laboratories can attain reproducible information. Previous optimization of multiphoton tissue imaging has been mainly performed in the context of single-modality imaging or simultaneous dual-modal imaging by SW-SHG&2PAF and LW-SHG&THG[1–3]. Here we attempt to optimize the excitation and detection conditions of SLAM microscopy.

The optimal excitation wavelength for SHG imaging was placed at around 900 nm for overall performance in signal strength, tissue penetration depth, and photodamage mitigation[4]. For 2PAF imaging, an excitation wavelength of 750 nm coupled with a blue detection band was optimized for imaging NADH, while an excitation wavelength of 900 nm coupled with an orange detection band was optimized for imaging FAD[5]. When THG imaging is considered, the excitation wavelength should lie within 1180-1350 nm to achieve optimal overall performance[6], although longer wavelengths may be needed to maximize the imaging depth[7]. In SLAM microscopy, we find that THG imaging limits the short-end of the excitation wavelength to ~1080 nm due to UV signal absorption, and 2PAF/3PAF imaging limits the long-end of the excitation wavelength to ~1170 nm due to low signal strength. Thus, the selected wavelength of 1110 nm within the two ends ensures an optimal imaging depth[8,9] with less risk of photodamage[10].

In addition to the excitation wavelength, another important parameter dictating the performance of SLAM microscopy is the duty cycle of the mode-locked source laser, i.e., the product of the pulse width and pulse repetition rate. A low duty cycle is favorable for generating a large signal[11], a deep imaging depth, and/or a good signal-to-background ratio[12]. This can be either realized by decreasing the pulse width[13] down to sub-15 fs pulses[14], or by decreasing the pulse repetition rate[12,15] down to 0.2 MHz[16]. However, slow imaging speeds (>1 pulse/pixel) and/or saturation of photon-counting photomultipliers limit the low-end of the repetition rate to ~1 MHz, while ambiguous separation of SHG/THG signals from 2PAF/3PAF signals limits the short-end of the pulse width to ~15 fs (corresponding to a transform-limited bandwidth of 100 nm @ 1100nm). Thus, a combination of a moderately short pulse width of 35 fs and a moderately low repetition rate of 10.2 MHz in SLAM microscopy reflects an optimal tradeoff.

For historical or practical reasons, the mode-locked lasers used for multiphoton imaging are dominated by a repetition rate f of ~80 MHz. However, there are reasons that a lower repetition rate is optimal for label-free multiphoton imaging. First, a decrease of f from 80 MHz to 10 MHz reduces the 2PAF (3PAF) saturation power $\langle I(t) \rangle$ from 30 mW (150 mW)

to 4 mW (19 mW)[17], which mitigates cell photodamage and plausible accumulated heating damage. Second, a theoretical study has shown that plasma-mediated nanocavitation dominates the biological photodamage up to f = 1 MHz, while free-electron-induced chemical decomposition or accumulated heating dominates the photodamage for f > 80 MHz[18]. Thus, the 10 MHz repetition rate may lie in a range where the two different biological photodamage mechanisms occur at higher thresholds. Third, the 10 MHz pulse repetition rate will be more beneficial than 80 MHz for fluorescence lifetime imaging of fluorophores with lifetimes longer than 12.5 ns, so that the accumulating photobleaching can be minimized. The combination of a relatively long excitation wavelength and a low duty cycle in SLAM microscopy allows for relatively fast imaging (up to a pixel rate of 250 kHz) at a relatively low average power (14 mW) with minimum risk of photodamage.

Supplementary Note 2: High-peak power fiber supercontinuum sources.

In two closely related studies[6,19], the low spectral power density and average power of the supercontinuum limited its peak power to excite the sample, so that a rather long pixel dwell time of 200 μs was used to perform label-free imaging and resulted in a rather slow imaging speed of 40 s/frame (380 x 380 pixels). In this study, we dramatically increase the peak power of the supercontinuum to allow for more than a two orders-of-magnitude increase in imaging speed. Given the source laser (femtoTrain) and a few test optical fibers, an aspheric lens with proper focal length (10-18 mm) and aperture (4-8 mm) was selected to couple the beam of the source laser into one fiber (Supplementary Table 1). The free-space-to-fiber laser coupling efficiency was above 80%, resulting in a high supercontinuum output power that was only limited by specific photodamage at the fiber entrance end. The supercontinuum output was collimated by a silver parabolic mirror (5 mm focal length). The hexagon shaped fundamental mode of photonic crystal fibers measured by a commercial spatial beam profiler was not distorted by the collimation optics. For comparison, we also used an ultrafast Yb:fiber laser (Fidelity, Coherent Inc., Santa Clara, USA) to generate supercontinuum from these fibers. This fiber laser emits 30-nJ 1070-nm 80-fs pulses at 71 MHz and an average power $\langle I(t)\rangle = 2.1$ W (Supplementary Table 1, Supplementary Fig. 8a-c).

We adopted the technique of "local pulse compression" in order to generate tunable 35 fs pulses across the spectral coverage of the fiber supercontinuum sources[20,21] at the focus of the super-apochromat microscope objective[22]. Somewhat surprisingly, this local pulse compression by multiphoton intrapulse interference phase scan (MIIPS)[23] failed in the fiber supercontinuum based on the 80-fs Yb:fiber laser (Source 4, Supplementary Fig. 8c; Supplementary Table 1), even though the supercontinuum was generated in the normal dispersion regime of the fiber[24]. We have tested other fibers (Supplementary Table 1) using this fiber laser, and none of the resulting supercontinuum sources supports the pulse compression. Because the highly-modulated supercontinuum spectral components close to the emission wavelength of the fiber laser are rather unstable (Supplementary Fig. 8c), we speculate that the amplification of the optical noise of the fiber laser during supercontinuum generation leads to temporal incoherence and prevent pulse compression. In contrast, local compression was successful for the supercontinuum sources based on the 10-MHz solid-state Yb laser (Sources 1-3, Supplementary Fig. 8a-c; Supplementary Table 1). The successful local pulse compression is characterized by the parallel lines in the MIIPS traces[23], with a significant increase of crystal SHG signal after pulse compression, and a Gaussian-like spectrum for the SHG signal (Supplementary Fig. 8a), leading to various tunable 35-fs sources with different tuning

ranges and peak powers (Supplementary Table 1). Source 1 was selected for the subsequent multiphoton imaging at 1110 nm due to its high peak power.

Interestingly, the local compression can also be largely attained by simply compensating the linear and quadratic chirps of the pulse[25], except that the compensating cubic phase is not mechanically attained using a grating (or prism) sequence but instead electronically imposed on the pulse shaper. The relaxation from 10-fs pulse compression[22] to 35-fs pulse compression allows the crystal SHG signal after the cubic phase compensation (quadratic compression) to approximate (within 10%) that after full phase compensation using MIIPS (Supplementary Fig. 8a). This eases the high-order phase distortion that requires full phase compensation in global pulse compression, so that the users avoid the experimental difficulty to work with short (<1 cm) fibers[24] and may save cost because simpler pulse shapers (e.g., prism- or grating-based compressor) without full phase compensation can be built and used.

In addition to supercontinuum generation, self-frequency shifted soliton generation (SSFS) has also been shown to generate similar high-peak power pulses (1150 nm after frequency doubling, 11.25 MHz, 20 nm in bandwidth) for combined SHG and THG imaging[26]. Despite the similarity of optical parameters, it may prove challenging to adapt the SSFS-based source to SLAM microscopy without major modifications, considering the significantly lower excitation efficiency for FAD/NADH at longer wavelengths as well as the relatively longer pulse duration (86 fs vs. 35 fs).

Supplementary Table 1. Evaluation and comparison of fiber supercontinuum sources.

	Source 1 (this study)	Source 2 (this study)	Source 3 (this study)	Source 4 (this study)	Source[20]	Source[6,19]
**Source laser**	femtoTrain	femtoTrain	femtoTrain	Fidelity	Custom	Custom
**Central λ**	1040 nm	1040 nm	1040 nm	1070 nm	1025 nm	1041 nm
**Pulse width τ**	314 fs	314 fs	314 fs	80 fs	170 fs	229 fs
**Rep. rate f**	10 MHz	10 MHz	10 MHz	71 MHz	44 MHz	80.2 MHz
**Avg. power <*I*(*t*)>**	3.5 W	3.5 W	3.5 W	2.1 W	2.1 W	2.5 W
**Fiber type**	PM-LMA-15	LMA-10	PM980	PM980	LMA-8	Custom
**Producer**	NKT photonics	NKT photonics	Nufern	Nufern	NKT photonics	NKT photonics
**Polarization maintaining**	Yes	No	Yes	Yes	No	Yes
**Mode field diameter**	12.6 µm @1064 nm	8.8 µm @1064 nm	6.6 µm @980 nm	6.6 µm @980 nm	7.5 µm @1064 nm	2.2 µm @1060 nm
**Zero dispersion wavelength**	1210 nm	1180 nm	~1300 nm	~1300 nm	1155 nm	None
**Fiber length**	25 cm	10 cm	22 cm	22 cm	8 cm	21 cm
**Supercontinuum output <*I*(*t*)>**	2.10 W	1.57 W	1.00 W	1.19 W	1.15 W	0.48 W
**Spectral coverage**	865-1210 nm	845-1200 nm	850-1290 nm	870-1300 nm	875-1190 nm	780-1320 nm
**Coherence**	full	full	full	partial	full	full
**After shaper <*I*(*t*)>**	0.63 W	0.47 W	0.28 W	0.35 W	0.31 W	0.14 W
**Shortest pulse width**	25 fs	10 fs	25 fs	N.A.	11 fs	25 fs
**<*I*(*t*)> for tunable 35-fs pulse**	40-160 mW	10-95 mW	15-60 mW	N.A.	20-80 mW	5-35 mW
**Tunable range for the 35-fs pulse**	910-1160 nm	880-1160 nm	910-1210 nm	N.A.	920-1150 nm	830-1250 nm
**Peak power for the 35-fs pulse**	160-640 KW	40-380 KW	60-240 KW	N.A.	20-80 KW	3-20 KW

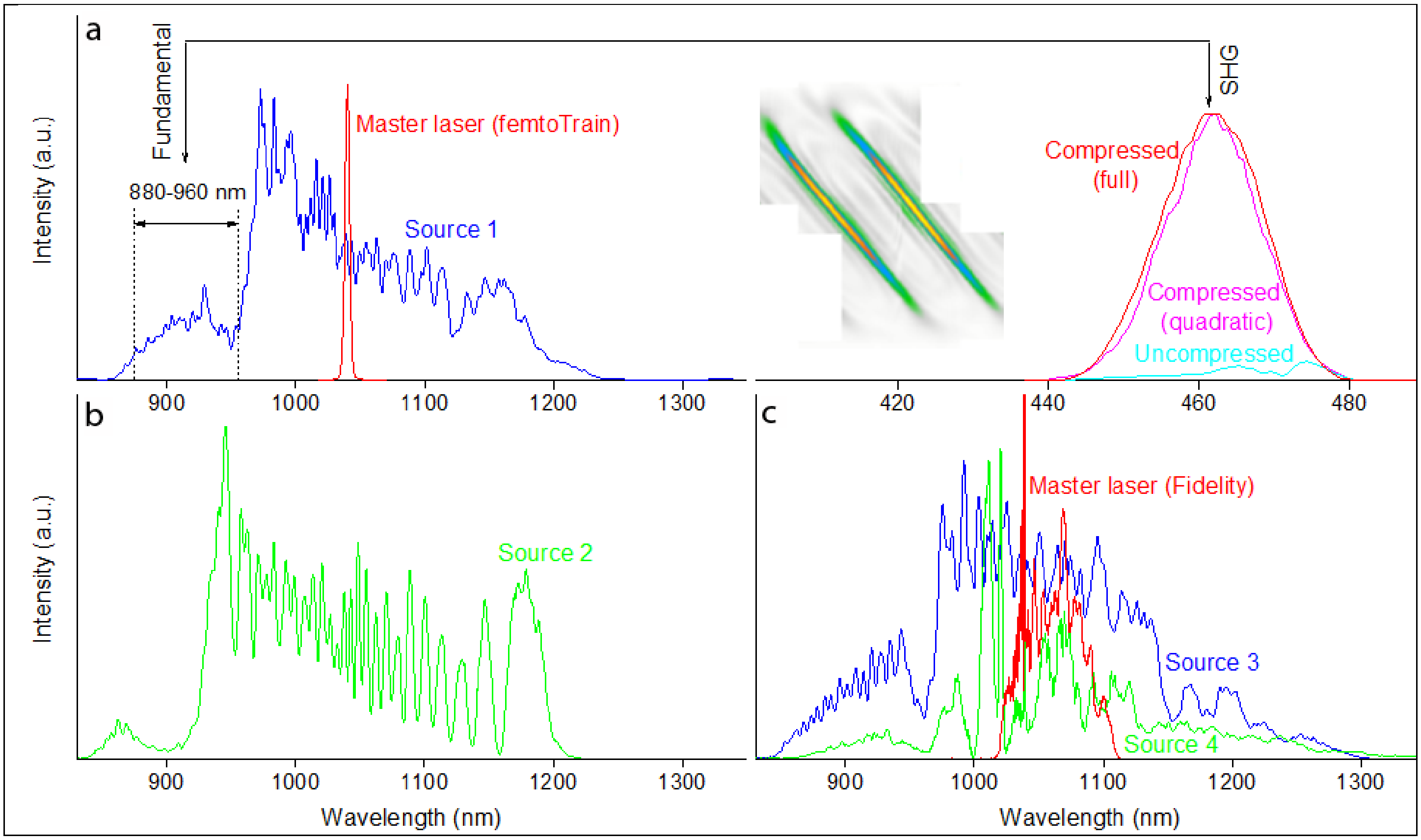

Supplementary Figure 8. (a) Spectrum of fiber supercontinuum Source 1 (blue curve, left) and master laser emission spectrum (red curve, left). The isolation of the 880-960 nm fundamental spectral component from Source 1 by amplitude shaping with the pulse shaper produces a crystal SHG spectrum without pulse compression (cyan curve, right), with full compression of MIIPS (red curve, right), and with quadratic compression of linear chirp -3500 fs^2 and quadratic chirp -35000 fs^3 (magenta curve, right). Inset: MIIPS trace of dual parallel lines indicative of transform-limited pulse compression. (b) Spectrum of fiber supercontinuum for Source 2. (c) Spectra of fiber supercontinuum for Source 3 and Source 4 and the emission spectrum of another master laser.

Supplementary Note 3: Correlation with anti-CD45 labelled images.

A CD45-based marker (Monoclonal antibody OX1, eBioscience) was injected into a living rat and acquired images of the tumor microenvironment before and after labelling (Supplementary Figure 5). Before labelling, the blue channel visualized mostly NADH-rich vesicles, while the yellow channel showed FAD-rich stroma cells and the purple channel picked up signals from THG-visible hollow spheres (6-8 μm in size), which are supposedly lymphocytes. After labelling, stained leukocytes should appear in the blue channel while the assignment for the other channels remain the same. As shown in Supplementary Figure 5, there are three groups of cells in this dataset: 1) yellow stroma cells that remain yellow after staining, which validates the specificity of both THG and CD45 for leukocytes; 2) leukocytes with overlapping THG and CD45 signals, which confirms the identify of these THG-visible cells; 3) leukocytes with CD45 but no THG signals, which suggests that SLAM microscopy only visualizes subpopulations of leukocytes. This might be due to the fact that THG is not highly efficient for detecting all lymphocytes as they carry few lipid granules. It could also be caused by technical issues, such as a slight change of the imaging depth before and after staining due to the injection procedure.

Supplementary References

1. Squier, J. & Müller, M. High resolution nonlinear microscopy: a review of sources and methods for achieving optimal imaging. *Rev. Sci. Instrum.* **72,** 2855–2867 (2001).
2. Hoover, E. E. & Squier, J. A. Advances in multiphoton microscopy technology. *Nat. Photonics* **7,** 93–101 (2013).
3. Xu, C. & Wise, F. W. Recent advances in fibre lasers for nonlinear microscopy. *Nat. Photonics* **7,** 875–882 (2013).
4. Chen, X., Nadiarynkh, O., Plotnikov, S. & Campagnola, P. J. Second harmonic generation microscopy for quantitative analysis of collagen fibrillar structure. *Nat. Protoc.* **7,** 654–69 (2012).
5. Huang, S., Heikal, A. A. & Webb, W. W. Two-Photon fluorescence spectroscopy and microscopy of NAD(P)H and flavoprotein. *Biophys. J.* **82,** 2811–2825 (2002).
6. Tu, H. *et al.* Stain-free histopathology by programmable supercontinuum pulses. *Nat. Photonics* **10,** 534–540 (2016).
7. Yildirim, M., Durr, N. & Ben-Yakar, A. Tripling the maximum imaging depth with third-harmonic generation microscopy. *J. Biomed. Opt.* **20,** 96013 (2015).
8. Balu, M. *et al.* Effect of excitation wavelength on penetration depth in nonlinear optical microscopy of turbid media. *J. Biomed. Opt.* **14,** 10508 (2009).
9. Kobat, D. *et al.* Deep tissue multiphoton microscopy using longer wavelength excitation. *Opt. Express* **17,** 13354 (2009).
10. Chen, I. H., Chu, S. W., Sun, C. K., Cheng, P. C. & Lin, B. L. Wavelength dependent damage in biological multi-photon confocal microscopy : a micro-spectroscopic comparison between femtosecond Ti:sapphire and Cr:forsterite laser sources. *Opt. Quantum Electron.* **34,** 1251–1266 (2002).
11. Zipfel, W. R., Williams, R. M. & Webb, W. W. Nonlinear magic: multiphoton microscopy in the biosciences. *Nat. Biotechnol.* **21,** 1369–1377 (2003).
12. Horton, N. G. *et al. In vivo* three-photon microscopy of subcortical structures within an intact mouse brain. *Nat. Photonics* **7,** 205–209 (2013).
13. Tang, S., Krasieva, T. B., Chen, Z., Tempea, G. & Tromberg, B. J. Effect of pulse duration on two-photon excited fluorescence and second harmonic generation in nonlinear optical microscopy. *J. Biomed. Opt.* **11,** 20501 (2006).
14. Xi, P., Andegeko, Y., Weisel, L. R., Lozovoy, V. V. & Dantus, M. Greater signal, increased depth, and less

photobleaching in two-photon microscopy with 10 fs pulses. *Opt. Commun.* **281,** 1841–1849 (2008).

15. Farrar, M. J., Wise, F. W., Fetcho, J. R. & Schaffer, C. B. *In vivo* imaging of myelin in the vertebrate central nervous system using third harmonic generation microscopy. *Biophys. J.* **100,** 1362–1371 (2011).

16. Theer, P., Hasan, M. T. & Denk, W. Two-photon imaging to a depth of 1000 µm in living brains by use of a Ti:Al_2O_3 regenerative amplifier. *Opt. Lett.* **28,** 1022 (2003).

17. Xu, C., Zipfel, W., Shear, J. B., Williams, R. M. & Webb, W. W. Multiphoton fluorescence excitation: new spectral windows for biological nonlinear microscopy. *Proc. Natl. Acad. Sci.* **93,** 10763–8 (1996).

18. Vogel, A., Noack, J., Hüttman, G. & Paltauf, G. Mechanisms of femtosecond laser nanosurgery of cells and tissues. *Appl. Phys. B Lasers Opt.* **81,** 1015–1047 (2005).

19. Tu, H. *et al.* Concurrence of extracellular vesicle enrichment and metabolic switch visualized label-free in the tumor microenvironment. *Sci. Adv.* **3,** e1600675 (2017).

20. Metzger, B., Steinmann, A. & Giessen, H. High-power widely tunable sub-20 fs Gaussian laser pulses for ultrafast nonlinear spectroscopy. *Opt. Express* **19,** 24354–60 (2011).

21. Liu, Y. *et al.* Suppressing short-term polarization noise and related spectral decoherence in all-normal dispersion fiber supercontinuum generation. *J. Light. Technol.* **33,** 1814–1820 (2015).

22. Tu, H., Liu, Y., Turchinovich, D. & Boppart, S. A. Compression of fiber supercontinuum pulses to the Fourier-limit in a high-numerical-aperture focus. *Opt. Lett.* **36,** 2315 (2011).

23. Lozovoy, V. V., Pastirk, I. & Dantus, M. Multiphoton intrapulse interference IV Ultrashort laser pulse spectral phase characterization and compensation. *Opt. Lett.* **29,** 775 (2004).

24. Tu, H. & Boppart, S. A. Coherent fiber supercontinuum for biophotonics. *Laser Photonics Rev.* **7,** 628–645 (2013).

25. Fork, R. L., Cruz, C. H., Becker, P. C. & Shank, C. V. Compression of optical pulses to six femtoseconds by using cubic phase compensation. *Opt. Lett.* **12,** 483–485 (1987).

26. Huang, J.-Y. *et al.* Fiber-based 1150-nm femtosecond laser source for the minimally invasive harmonic generation microscopy. *J. Biomed. Opt.* **22,** 36008 (2017).
