# Supplementary material for: Intravital imaging by simultaneous label-free autofluorescence-multiharmonic microscopy
Source: Nat Commun. 2018 May 29;9:2125. doi: 10.1038/s41467-018-04470-8 (PMC5974075; doi:10.1038/s41467-018-04470-8)
Supplement: Supplementary file 3 — Description of Additional Supplementary Files [file 41467_2018_4470_MOESM3_ESM.pdf]

Description of Additional Supplementary Files

File Name: Supplementary Movie 1
Description: Volumetric image stack from 40 µm to 115 µm below the surface. Recording frame rate: 0.5 f.p.s. Playback at 30 f.p.s. Data correspond to Fig. 2f-h.

File Name: Supplementary Movie 2
Description: Neutrophil slow rolling along the vessel wall. Recording duration:15 min. Recording frame rate: 0.5 f.p.s. Playback at 30 f.p.s. Data correspond to Fig. 3a.

File Name: Supplementary Movie 3
Description: Leukocyte adhering to and crawling along the vessel wall. Recording duration: 27 min. Recording frame rate: 0.5 f.p.s. Playback at 30 f.p.s. Data correspond to Fig. 3b.

File Name: Supplementary Movie 4
Description: Leukocyte swarming (raw data). Recording duration: 70 min. Recording frame rate: 0.5 f.p.s. Playback at 100 f.p.s. Data correspond to Fig. 4.

File Name: Supplementary Movie 5
Description: Leukocyte swarming with traces (after smoothing). Recording duration: 70 min. Recording frame rate: 0.5 f.p.s. Playback at 100 f.p.s. Data correspond to Fig. 4
